# Post-Quantum Secure Identity-Based Signature Scheme with Lattice Assumption for Internet of Things Networks

**DOI:** 10.3390/s24134188

**Published:** 2024-06-27

**Authors:** Yang Zhang, Yu Tang, Chaoyang Li, Hua Zhang, Haseeb Ahmad

**Affiliations:** 1 College of Food and Bioengineering, Zhengzhou University of Light Industry, Zhengzhou 450001, China; 2 College of Software Engineering, Zhengzhou University of Light Industry, Zhengzhou 450001, China; 3 Department of Computer Science, National Textile University, Faisalabad 37610, Pakistan

**Keywords:** Internet of Things, identity-based signature, post-quantum, lattice cryptography

## Abstract

The Internet of Things (IoT) plays an essential role in people’s daily lives, such as healthcare, home, traffic, industry, and so on. With the increase in IoT devices, there emerge many security issues of data loss, privacy leakage, and information temper in IoT network applications. Even with the development of quantum computing, most current information systems are weak to quantum attacks with traditional cryptographic algorithms. This paper first establishes a general security model for these IoT network applications, which comprises the blockchain and a post-quantum secure identity-based signature (PQ-IDS) scheme. This model divides these IoT networks into three layers: perceptual, network, and application, which can protect data security and user privacy in the whole data-sharing process. The proposed PQ-IDS scheme is based on lattice cryptography. Bimodal Gaussian distribution and the discrete Gaussian sample algorithm are applied to construct the fundamental difficulty problem of lattice assumption. This assumption can help resist the quantum attack for information exchange among IoT devices. Meanwhile, the signature mechanism with IoT devices’ identity can guarantee non-repudiation of information signatures. Then, the security proof shows that the proposed PQ-IDS can obtain the security properties of unforgeability, non-repudiation, and non-transferability. The efficiency comparisons and performance evaluations show that the proposed PQ-IDS has good efficiency and practice in IoT network applications.

## 1. Introduction

The Internet of Things (IoT) plays a very essential role in people’s daily production and life [1]. As daily travel, consumption, production, medical treatment, etc., are gradually becoming digital and intelligent, a variety of smart IoT devices surround people’s lives. Although these IoT devices provide convenience, they also bring many security threats to people’s lives and property safety. IoT network security is gaining much attention with the IoT network development [2].

**Figure 1:**
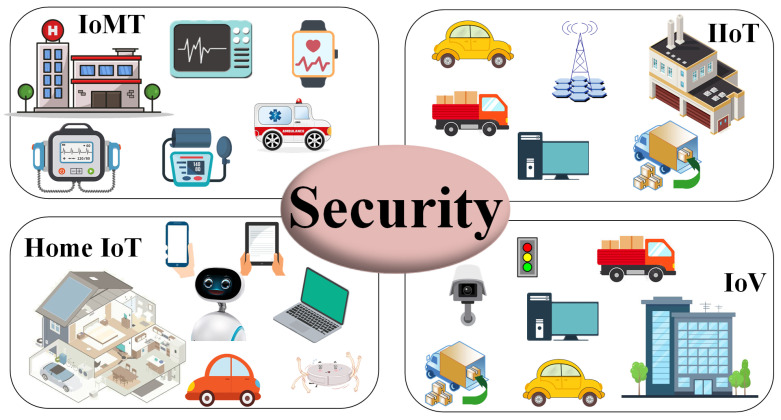
Security issues for IoT network.

The security issue is the most essential part of data sharing among different IoT devices through the public network. As shown in Figure 1, data security and user identity privacy should be given more consideration in IoT network applications, such as the Internet of Medical Things (IoMT) [3], the Home Internet of Things (Home IoT) [4], the Internet of Vehicles (IoV) [5], and the Industrial Internet of Things (IIoT) [6]. In IoMT systems, medical data are essential in medical diagnosis, drug development, disease prediction, and medical device creation. The security of medical data and user privacy is the guarantee for data value play. In the Home IoT, smart home devices collect data about the user’s daily life, health condition, living habits, and personal privacy. These data are related to the safety of users’ lives and property. In IoV systems, smart driving cars, cameras, and traffic lights collect information about roads, traffic, logistics, and more in real time. Infrastructure information, such as geography and transportation, is relevant to national security. In IIoT systems, people, machines, materials, materials, law, environment, enterprise upstream and downstream, products, users, and other elements are connected to achieve data transmission between elements. It provides identity data acquisition, label management, identity registration, identity analysis, data processing, and identity data modeling functions to achieve the marking, management, and positioning of elements, and the industrial Internet identity resolution security mechanism has become the basis of industrial Internet applications. IoT data are becoming a more important asset for IoT network applications, which plays an essential role in process improvement, industrial upgrading, and social progress. Data security and user privacy security are two main parts of most IoT network applications that should be given more consideration.

Blockchain is a distributed data management technology that utilizes the consensus mechanism and cryptographic algorithm to achieve secure data sharing in different and strange modes [7]. The consensus mechanism helps the system achieve distributed consensus with a uniform ledger for the whole network, for example, proof of work (PoW) [8], proof of stake (PoS) [9]. The cryptographic algorithm helps the system achieve, for example, DES [10], ECC [11]. With the deepening of the application of blockchain technology in IoT network applications, there emerge many different kinds of chains with different distributed structures, consensus protocols, and cryptographic algorithms. These chains bring the IoT networks back to a centralized model as the IoT data cannot be freely shared among different IoT devices. So, cross-chain technology has gained more attention in recent years.

Cryptography is a general technology that utilizes difficult mathematical problems to achieve secure data sharing among public Internet work [12]. However, the development of quantum algorithms threatens current classical cryptographic algorithms-based IoT network applications [13]. Lattice cryptography is a promising anti-quantum theory that utilizes lattice assumptions to improve post-quantum security [14]. For example, the Number Theory Research Unit (NTRU) consists of the convolution of polynomials, which is based on the shortest vector problem (SVP) on a lattice theory [15]. It uses truncated polynomial rings to encrypt and decrypt data. GGH is an asymmetric cryptographic algorithm constructed by the nearest vector problem [16]. Learning With Errors (LWE) is an unsolvable problem in machine learning that can be reduced to a SIVP lattice problem [17]. There is a variant of ring-based LWE where polynomials replace the integer vectors in the LWE problem. The identity-based signature mechanism utilizes the identity of IoT devices to establish the traceability mechanism for IoT data sharing.

This paper focuses on the security issues of data loss, privacy leakage, and anti-quantum weakness in most current IoT network applications, and proposes a PQ-IDS to improve the data-sharing security among different IoT devices. The main contributions of this paper are summarized as follows.

1. A security model is constructed with blockchain and the PQ-IDS algorithm for IoT network applications. This model divides the IoT network into three layers: perceptual, network, and application, which help to establish a distributed and secure data-sharing mechanism for IoT network applications.
2. A PQ-IDS scheme is proposed based on lattice assumption, and it inserts the IoT device’s identity into the signature scheme to achieve the traceability of IoT data sharing among the IoT network. This scheme helps to achieve anti-quantum attack security with the lattice cryptography theories.
3. Security analysis and proof about the correctness, unforgeability, non-repudiation, traceability, and post-quantum security are presented. The performance evaluations of key-size data-sharing transactions are presented. These results show the efficiency and practicality of the proposed security model and PQ-IDS scheme.

Next, Section 2 gives some related work, Section 3 gives some preliminaries, Section 4 gives the PQ-IDS scheme, Section 5 gives the security proof of the PQ-IDS scheme, Section 6 gives the efficiency comparison and performance, and Section 7 gives the conclusion.

## 2. Related Work

### 2.1. IoT Network Security

In IoT network applications, there exist a lot of attacks, such as deception, information leakage, tampering, denial of service (DoS), and privilege escalation, which threaten the personal and property safety of the system users. For the deception attack, Rehman et al. [18] utilized the model-based method to construct a proactive defense framework, which aggregated the technologies of proactive defense mechanisms and cyber deception to resist the deception attack in IoT networks. For the information leakage, Xu et al. [19] established a data-sharing framework with blockchain technology for IIoT, and inserted federated learning into this scheme to improve the tamper-proof and decentralized capabilities. Singh et al. [20] constructed a privacy-preserving model for IoMT with blockchain and zero-knowledge proof, which strengthened the adaptability in lightweight computer devices. For the tampering, Zhang et al. [21] introduced a malware traffic classification model dependent on neural architecture search, which could improve the searchability of the optimal model in a realistic IoT environment. Li et al. [22] designed a hierarchical and multi-group data-sharing scheme for IIoT, which could improve the data access control ability against the key leakage resilience attack. For the DoS, Malik et al. [23] introduced a detect model based on feature engineering and machine learning, which achieved resisting distributed DoS in standardized IoT. Khanday et al. [24] proposed an intrusion detection platform based on machine learning and deep learning classifiers for IoT networks, which could improve the detection accuracy and efficiency of DDoS attacks. For the privilege escalation, Mehmood et al. [25] constructed an insider threat detection and classification framework based on machine learning, and introduced a systematic approach to resist the privilege escalation in relation to the anomalies and security problems. However, the methods used in these references are all classical cryptographic algorithms, which cannot resist quantum attacks. This paper plans to utilize blockchain technology and a post-quantum signature algorithm to provide anti-quantum security for data-sharing in IoT networks.

### 2.2. Signatures for IoT Networks

Digital signatures play an essential role in the data-sharing processes in different IoT network applications. In particular, the identity-based signature (IDS) has widespread application due to its identification mechanisms and traceability. Liu et al. [26] designed a distributed multi-signature scheme with discrete logarithms to improve the security and efficiency of IoT identification with centralized signature schemes. Jia et al. [27] designed a certificateless signature protocol with an ECC-based discrete logarithm problem, and stated that it was more suitable for resource-limited IoT devices. Du et al. [28] figured out the security weakness of the scheme in Ref. [27], and presented a new signature scheme with the ECC cryptosystem, which could resist the forger attack from the super adversaries. Li et al. [29] gave an aggregate signature scheme to strengthen the security of large-scale data transmission in IIoT, which also utilized blockchain technology to achieve secure, distributed, and autonomous data management. Bao et al. [30] presented an identity management scheme to protect identity privacy for IIoT, and took the blockchain technology together to achieve distributed privacy-preserving.

In most current IoT network applications, the IDS schemes constructed by large integer factorization and discrete logarithm problems have good applicability to satisfy the needs of IoT devices with high throughput and low latency. However, these IDS schemes cannot resist the quantum attack [31]. Therefore, post-quantum cryptography has gained more attention in current academia and industry to improve the security properties of anti-quantum attacks. Lattice cryptography is one promising post-quantum algorithm. Srivastava et al. [32] presented an identity-based multi-signature scheme with a multivariate quadratic problem, which could achieve anti-quantum security for real-time information exchange in IoV. Wang et al. [33] introduced a proxy signature scheme with lattice assumption, which utilized the lattice basis delegation and preimage sample technologies to achieve post-quantum secure IoT. Wu et al. [34] designed an ID-based proxy signature based on the NTRU lattice and utilized message recovery technology to guarantee secure message exchange among different IoT devices. Prajapat et al. [35] gave an ID-based aggregate signature based on lattice cryptography, which could improve the messages promptly, vehicle identity confidentiality, and authentication swiftly in the vehicle ad hoc networks (VANETs). Sun et al. [36] utilized the path signature to construct an XAI-enabled network, which could achieve efficient time series classification. The simple comparisons of these former schemes are shown in the following Table 1. Although these lattice-based IDS schemes strengthen the anti-quantum ability of IoT networks, there still exist some security issues of privacy leakage, big key size, and high-performance consumption. This paper plans to design a post-quantum secure IDS for general IoT network applications.

## 3. Preliminaries

### 3.1. Lattice Theory

Some definitions of the lattice theories are presented, which are in relation to the construction of the DVS scheme. First and foremost, some notations of parameters used in this paper are explained in Table 2.

**Definition 1.**  ***Lattice* [14]** *: Given a set of linearly independent vectors $v_{1},\cdots ,v_{n}\in R^{m}$, the lattice $\Lambda_{L}$ is defined as Equation (1) as follows:*

(1) $$\Lambda_{L}=\left\{a_{1}v_{1}+a_{2}v_{2}+\cdots +a_{n}v_{n}:a_{1},a_{2},\cdots ,a_{n}\in Z\right\}$$

*The matrices $A=\left(a_{1},\cdots ,a_{m}\right)\subset R^{n\times m}$ establish a basis for the lattice* Λ *with dimension n and rank m. In general, n and m satisfy m=O(nlogq).*

**Definition 2.**  ***q-ary Lattice* [14]** *“q-ary” lattice is defined with a prime number q, matrix $A\in Z_{q}^{n\times m}$, which is shown in Equation (2) as follows:*

(2) $$\begin{array}{cc} \; & L^{⊥}\left(A\right)=\left\{x\in Z^{m}|Ax=0\;modq\right\}\\ \; & L_{y}^{⊥}\left(A\right)=\left\{x\in Z^{m}|A^{T}y=x\;mod\;q\;for\;y\in Z^{n}\right\} \end{array}$$

**Definition 3.**  ***Gaussian distribution * [37]** *: With standard deviation σ∈R and center c∈R evaluated at x∈R, the Gaussian distribution is defined by $\rho_{c,\sigma }\left(x\right)=exp\left(\frac{-{\left(x-c\right)}^{2}}{2\sigma^{2}}\right)$, and more generally by $\rho_{c,\sigma }\left(x\right)=exp\left(\frac{-||x-{c||}^{2}}{2\sigma^{2}}\right)$ for $x,c\in R^{n}$. Here, $\rho_{\sigma }\left(x\right)$ represents the Gaussian distribution when the center c=0. $D_{\sigma }\left(x\right)=\rho_{\sigma }\left(x\right)/\rho_{\sigma }\left(Z\right)$ represents the discrete Gaussian distribution over Z with center c=0. $D_{\sigma }\left(x\right)=\rho_{\sigma }\left(x\right)/\rho_{\sigma }\left(Z^{m}\right)$ represents the more general situation over $Z^{m}$ with center c=0.*

**Definition 4.**  **$ℜ-{\mathrm{SIS}}_{q,n,m,\beta }^{\kappa }$ *problem*  [37]** *: Given ring ℜ, a distribution κ over ${ℜ}_{q}^{n*m}$, $ℜ-SIS_{q,n,m,\beta }^{\kappa }$ is a problem to find non-zero $v\in {ℜ}_{q}^{m}$ to achieve Equation (3), as follows:*

(3) Av=0

where $A\in {ℜ}_{q}^{n*m}$, and ${\left||v|\right|}_{2}\leq \beta$.

### 3.2. Model Definitions

(1) Scheme model

For an ID-based signature, there are four probabilistic polynomial time algorithms: Setup, KeyExt, Sign, and Verify.

- **Setup($1^{n}$):** Given a security parameter *n* and parameters *q* and *m*, KGC derives some public parameters pp and the master key msk.
- **KeyExt(pp,msk):** Given the public parameters pp and the master key msk, KGC generates public/private key pairs (pk,sk) for the system user with identity $ID_{i}$.
- **Sign(pp,sk,μ):** The signer generates a signature *e* in relation to a with message μ with his own private key sk.
- **Verify(pp,pk,μ,e):** The verifier verifies the *e*’s legality with pp and pk, and output Accept/Reject.

(2) Security model

To prove the security of this ID-based signature, it generally establishes a query–response game in a random oracle model. By making reasonable assumptions about the adversary *A* in the game, it can derive a contradiction that does not correspond to reality by utilizing the proof by contradiction. Then, it will derive that the ID-based signature is secure. Next are the detailed descriptions of this security model.

- *Initialize: C* executes the **Setup** algorithm to derive the system pp.
- *Query*: *A* queries the Hash, secret key, and signature algorithms with enough times in polynomial time, respectively.
  − *Hash query*: *A* queries all hash algorithms about the user $ID_{i}$ or message $\mu_{j}$ (not the target user $ID_{i}^{*}$ and message $\mu_{j}^{*}$).
  − *Secret key query*: *A* queries the private key about the user $ID_{i}$ (not the target user $ID_{i}^{*}$).
  − *Signature query*: *AA* queries the signature about the user $ID_{i}$ or message $\mu_{j}$ (not the target user $ID_{i}^{*}$ and message $\mu_{j}^{*}$).
- *Forge*: *A* generates a signature (e,μ) about the target user $ID_{i}^{*}$ and message $\mu_{j}^{*}$, and sends it to *C*.
- *Challenge*: *C* generates another signature $\left(e^{\prime },\mu^{\prime }\right)$ about the target user $ID_{i}^{*}$ and message $\mu_{j}^{*}$ based on the hypothesis. Then, *C* attempts to derive a solution for the $Z-SIS_{q,n,m,\beta }^{\kappa }$ instance by utilizing these two signatures (e,μ) and $\left(e^{\prime },\mu^{\prime }\right)$.
- *Analyze*: The lattice assumption SIS cannot be solved with the current highest computing power. This implies that the hypothesis of the former adversary *A* is invalid.

## 4. PQ-IDS for IoT Network Applications

This section first introduces a security model for IoT network applications and then designs a post-quantum identity-based signature (PQ-IDS) scheme with the lattice assumption.

### 4.1. Security Model for IoT Network Application

**Figure 2:**
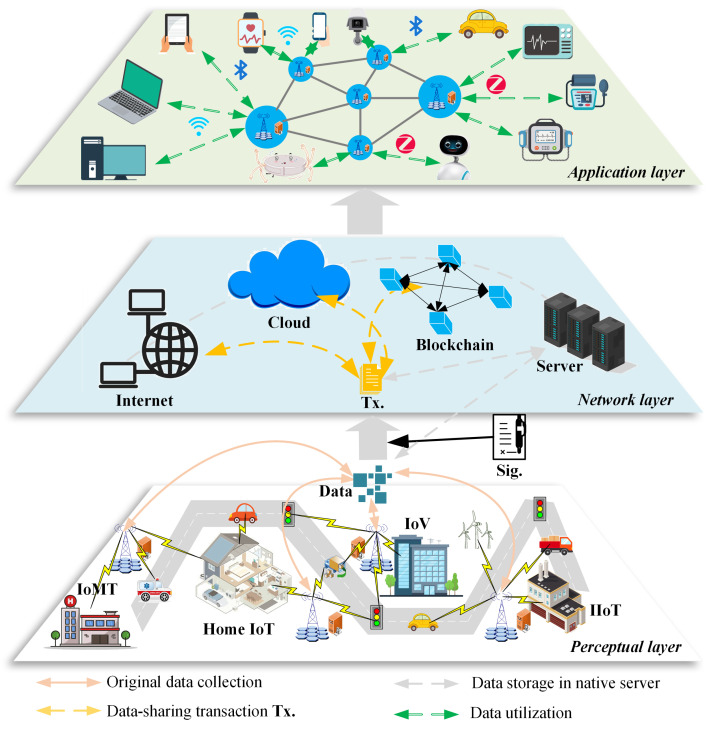
IoT network security model.

As the former described in Section 1, the security issue is an essential part of information communication and data exchange processes in every IoT application, such as IoMT, Home IoT, IoV, and IIoT. This section divides the IoT network into three layers: perceptual, network, and application (as shown in Figure 2), and gives detailed descriptions of these three layers, security demands, and solutions.

- *Perceptual layer*: This layer is a data collection process. From different smart IoT devices, many types of data are selected. These data mainly appear in text, figure, and video forms. As in the IoMT environment, the medical data generally contain health data collected from wearable smart devices, diagnosis data created by patients and doctors, testing data generated by medical devices, and other related data about health insurance, banking, etc. In the Home IoT environment, the data are collected from smart home devices, such as door locks, cameras, refrigerators, washing machines, sweepers, smart speakers, air purifiers, etc. As in the IoV environment, the data are collected from smart vehicles, road detection, cameras, traffic lights, etc. In the IIoT environment, the data are collected from logistics trucks, wind-generating sets, and other related industrial devices. These collected data are signed with the signatures of IoT devices or device owners. The identities inserted in these signatures guarantee the data verifiability and traceability by utilizing the PQ-IDS scheme.
- *Network layer*: This layer is a data transmission process. The selected data are transmitted through the public network, such as the Internet, cloud, or blockchain. Under the help of communication protocol, these data are transmitted into the transaction Tx, and they also contain the signatures of IoT devices or device owners. Through those public networks, these transactions go to the node that needs them. Meanwhile, it needs a server with enough storage space for data storage and management. The Internet and cloud networks are generally public, which cannot provide a strong security guarantee for cross-domain data sharing. Blockchain technology changes the traditional centralized form to a distributed form, which improves data-sharing transparency and cross-institution data-sharing ability. As in blockchain-based IoT networks, the system transaction verification also can be executed by the PQ-IDS to achieve network-wide consistency. Some security issues of data tampering, malicious theft, and other network attacks generally occur in this layer.
- *Application layer*: This is a data-utilizing process. A number of IoT devices are included in the IoT network, which continuously performs data exchange and processing. The collection, storage, calculation, and analysis of massive data support different IoT application purposes for people’s daily lives. For example, IoV data can solve some complex problems in traffic. On the one hand, the data can help understand the traffic flow situation, and improve the driving route for the vehicle owner; On the other hand, the data can also provide decision making for the traffic department in the aspects of road planning, real-time scheduling of signal lights, and vehicle diversion. More importantly, the PQ-IDS scheme plays an essential role in guaranteeing data credibility. This is because the operations throughout the life cycle of the data are recorded, and the identity information of all operators can be traced through signature verification.

In order to trace the data source and find the IoT fault point, it is imperative to label the identity of the operator in the processes of data generation, transmission, processing, and use. Identity-based signatures can guarantee security, integrity, and non-repudiation, which can help to achieve IoT data secure transmission and utilization. Meanwhile, current IoT network-based information systems are equated with traditional cryptographic algorithms that cannot provide security in the quantum computer age. This paper designs a post-quantum identity-based signature (PQ-IDS) scheme with lattice assumption, which can cover the security demands for data-sharing among IoT devices in different layers of the IoT network.

### 4.2. Details of PQ-IDS

To improve the privacy security of data-sharing transactions in BIoMT, an identity-based signature scheme with lattice assumption has been given. Next, this scheme mainly contains four algorithms.

$\mathrm{Setup}\;(1^{n})$: Given the security parameter *n*, a prime number *q* with q=q(n)≥3, a positive integer *m* with m≥5nlogq, $L=O(\sqrt{nlogq})$, and $\sigma =L\cdot \omega (\sqrt{logn})$, the KGC first derives the system parameters and master private key. Details of this algorithm are presented in Algorithm 1.
**Algorithm 1** Setup**Input:** Security parameter *κ*, a prime number *q*, a positive integer *m***Output:** Public parameter *pp* and master key *msk*
1: KGC derives an approximate random distribution matrix $mpk=A\in Z_{q}^{n\times m}$ as the master public key, and a basis $T\in Z_{q}^{m\times m}$ from $\Lambda^{⊥}\left(A\right)$, which satisfy $\left|\right|\tilde{T}\left|\right|\leq L$;2: Selects two hash functions $H_{1}:{\left\{0,1\right\}}^{*}\to Z_{q}^{n},H_{2}:{\left\{0,1\right\}}^{*}\to Z_{q}$;3: Serves msk=T;4: Outputs $pp=\{A,H_{1},H_{2}\}$, and keeps msk=T secretly.

$\mathrm{KeyExt}\;(ID_{i},msk,pp)$: Given the system user’s identity $ID_{i}$, KGC generates the key pairs (pk,sk) in relation to $ID_{i}$. Details of this algorithm are presented in Algorithm 2.
**Algorithm 2** KeyExt**Input:** pp, msk, and system user’s identity $ID_{i}$**Output:** Key pairs (pk,sk)1: KGC computes $a_{ID_{i}}=H_{1}\left(ID_{i}\right)\in Z_{q}^{n}$;2: Computes $s_{ID_{i}}\leftarrow Samplepre\left(A,T,a_{ID_{i}},\sigma \right)\in Z_{q}^{m}$, where $\sigma \geq \left|\right|\tilde{T}\left|\right|\omega \left(\sqrt{logm}\right)$, $a_{ID_{i}}modq=A\cdot s_{ID_{i}}$, and $\left|\right|s_{ID_{i}}\left|\right|\leq \sigma \sqrt{m}$;3: Outputs the public key $pk=a_{ID_{i}}$ and secret key $sk=s_{ID_{i}}$ for system user with $ID_{i}$.

Sign(pp,pk,sk,μ): Given the message μ, the signer utilizes his secret key sk to sign on the data-sharing transaction. Details of this algorithm are presented in Algorithm 3.
**Algorithm 3** Sign**Input:** Message μ, system public key *A*, signer’s private key $s_{ID_{i}}$**Output:** Signature *e*1: The user $ID_{i}$ randomly selects $x\in D_{\sigma }^{m}$;2: Computes $c=H_{2}\left(Ax,\mu \right)$;3: Computes $e=s_{ID_{i}}c+x$;4: Output the signature <e,c> with probability $min(\frac{D_{\sigma }^{m}\left(e\right)}{M_{2}D_{S\mu ,\sigma }^{m}\left(z\right)},1)$; otherwise, restart.

$\mathrm{Verify}(pp,\mu ,a_{ID_{i}},<e,c>)$: The verifier utilizes the system parameter pp and $pk=a_{ID_{i}}$ to verify the legality of <e,c> in relation to message μ. Details of this algorithm are presented in Algorithm 4.
**Algorithm 4** Verify**Input:** μ, $a_{ID_{i}}$, and <e,c>**Output:** Reject or accept1: **if** ||e||>L **then**2:    Reject it3: **end if**4: **if** ${\left||e|\right|}_{\infty }>q/4$ **then**5:    Reject it6: **end if**7: If $c=H_{2}\left(Ae-a_{ID_{i}}c\;mod\;q,\mu \right)$, accept; otherwise, reject.

This PQ-IDS scheme establishes data verification, identity authentication, and data-traceable mechanisms for IoT data sharing. Meanwhile, the lattice assumption guarantees the anti-quantum attack property for IoT network applications.

### 4.3. Example Application

**Figure 3:**
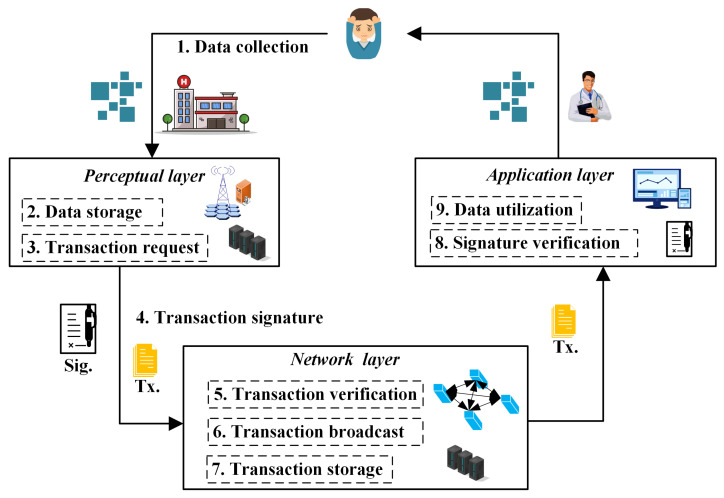
Data-sharing transaction through the blockchain-based IoMT.

This subsection presents an example application analysis of the former proposed security model and PQ-IDS scheme into the blockchain-based IoMT system. This signature scheme acts as two roles in the blockchain transactions in the IoMT system: the transaction signature and system transaction verification. Under the proposed security model, the data-sharing transaction through the blockchain-based IoMT system can be divided into the following six steps, also shown in the following Figure 3.

- *Data collection*: Through smart medical devices, the daily health data and hospital test data are collected and uploaded to the IoMT system. To establish a traceability mechanism, the medical data are labeled with the signatures of related IoMT devices or operators.
- *Data storage*: In the blockchain-based IoMT system, the real data are stored in the native server. The storage address, operation records, and other related lightweight information are uploaded into the blockchain ledger.
- *Transaction request*: When one patient wants to authorize the doctor to view their health data, they execute a transaction request. This situation is a process of seeing a doctor or scientific research as one user attempts to share data with the other system user. This transaction only sends authorization for these data and the target user must pass this authentication before they can access control on the real medical data.
- *Transaction signature*: Based on the former proposed PQ-IDS scheme, this patient signs the transaction with their private key sk. This signature is a proof that other users confirm the data’s validity by opening it. Meanwhile, this patient cannot deny this signature when some medical disputes occur.
- *Transaction verification*: This is the process of verifying the consistency of blockchain transactions across the network. The packaged transactions are sent to the verification nodes who check the legality of the transactions and sign them. The verification process is according to the consensus protocol proof of work (PoW) or proof of stake (PoS).
- *Transaction Broadcast*: The verified transactions will be broadcast to all the nodes in the IoMT network. This process mainly synchronizes the ledger across the network, but it also needs to obtain the access control rights of the real data before the target user can view the original data.
- *Transaction storage*: These transaction data belong to lightweight information, which is recorded in the blockchain ledger. This blockchain ledger is public and immutable, guaranteeing the transaction data’s security and traceability.
- *Signature verification*: The target user (doctor) first verifies the signature of the transaction originator.
- *Data utilization*: These medical data can be used for disease diagnosis, drug discovery, medical research, and medical device development.

In general, the proposed security model with a PQ-IDS scheme provides a fundamental framework for data-sharing in the blockchain-based IoMT system. It is also suitable for privacy protection in the data-sharing processes in other IoT network applications.

## 5. Security Analysis

This security analysis of the PQ-IDS scheme is given in this section. The correctness analysis is given first, and then the unforgeability, non-repudiation, and non-transferability are proved in the random oracle model under the principle defined in the former section.

### 5.1. Correctness

As shown in Algorithm 4, when the signature satisfies ||e||>L or ${\left||e|\right|}_{\infty }>q/4$, the signature <e,c> is valid iff the following equation Equation (4) holds.

(4) $$H_{2}\left(Ae-a_{ID_{i}}c\;modq,\mu \right)=H_{2}\left(Ax,\mu \right)$$

Detailed processes of the equation $Ae-a_{ID_{i}}c=Ax\;modq$ are shown in Equation (5) as follows:

(5) $$\begin{array}{cc} Ae-a_{ID_{i}}c & =A\left(s_{ID_{i}}c+x\right)-a_{ID_{i}}c\\ \; & =As_{ID_{i}}c+Ax-a_{ID_{i}}c\\ \; & =a_{ID_{i}}c+Ax-a_{ID_{i}}c\\ \; & =Ax\;modq \end{array}$$

### 5.2. Unforgeability

**Theorem 1.**  *This PQ-IDS is secure against the forgery attack as this signature cannot be forged under the hardness of SIS problem $Z-SIS_{q,n,m,\beta }^{\kappa }$.*

**Proof.**  A query–response game is established first in which an adversary *A* acts as the query member, and a challenger *C* acts as the response member. Then, these two members execute this game to prove that this PQ-IDS can capture the security property of unforgeability. In this game, *A* attempts to obtain enough signature information (without the target message $\mu^{*}$ and user $ID_{i}^{*}$) and has the ability to create a valid signature. *C* responds to *A*’s queries according to the signature scheme construction, and attempts to solve a $Z-SIS_{q,n,m,\beta }^{\kappa }$ instance with the knowledge of the forged signature in polynomial time. Next are the detailed steps of the query–response game. □

- *Initialize*: *C* performs the **Setup** algorithm to derive (n,m,q,k,σ).
- *Query*: *A* queries the Hash, secret key, and signature algorithms with enough times in polynomial time, respectively.
  − *$H_{1}$ query*: *A* queries the hash function $H_{1}$ about the user’s identity $ID_{i}$. Next, *C* keeps a list $L_{H_{1}}$ to store these query results. When they obtain the queries from *A*, *C* first checks the list $L_{H_{1}}$ whether the result pair $\left(a_{ID_{i}},ID_{i}\right)$ exists or not. If yes, they send $a_{ID_{i}}$ to *A* as the response of $H_{1}$’s query about $ID_{i}$. If no, they compute $a_{ID_{i}}=H_{1}\left(ID_{i}\right)$, send $a_{ID_{i}}$ to *A*, and record $\left(a_{ID_{i}},ID_{i}\right)$ in the list $L_{H_{1}}$. Here, *A* can execute this query with enough times $q_{H_{1}}$ about different identities $\left\{ID_{1},ID_{2},...,ID_{q_{H_{1}}}\right\}$.
  − *$H_{2}$ query*: *A* queries the hash function $H_{2}$ about the message $\mu_{i}$ (is not the target message $\mu^{*}$). Next, *C* keeps a list $L_{H_{2}}$ to store these query results. When they obtain the queries from *A*, *C* first checks the list $L_{H_{2}}$ whether the result pair $\left(c_{i},\mu_{i}\right)$ exists or not. If yes, they send $c_{i}$ to *A* as the response of $H_{2}$’s query about $I\mu_{i}$. If no, they randomly choose $x\in D_{\sigma }^{m}$, compute $c=H_{2}\left(Ax,\mu_{i}\right)$, send $c_{i}$ to *A*, and record $\left(c_{i},\mu_{i}\right)$ in the list $L_{H_{2}}$. Here, *A* can execute this query with enough times $q_{H_{2}}$ about different messages $\left\{\mu_{1},\mu_{2},...,\mu_{q_{H_{2}}}\right\}$.
  − *Private key query*: *A* queries the private key of the user $ID_{i}$ (is not the target user $ID_{i}^{*}$). Next, *C* keeps a list $L_{P}$ to store these query results. When they obtain the queries from *A*, *C* first checks the list $L_{P}$ whether the result pair $\left(s_{ID_{i}},ID_{i}\right)$ exists or not. If yes, they send $s_{ID_{i}}$ to *A* as the response of a private key query about $ID_{i}$. If no, they compute $s_{ID_{i}}\leftarrow Samplepre\left(A,T,a_{ID_{i}},\sigma \right)\in Z_{q}^{m}$, sends $s_{ID_{i}}$ to *A*, and records $\left(s_{ID_{i}},ID_{i}\right)$ in the list $L_{P}$. Here, *A* can execute this query with enough times $q_{P}$ about different identities $\left\{ID_{1},ID_{2},...,ID_{q_{P}}\right\}$.
  − *Signature query*: *A* queries the signature of the user $ID_{i}$ about the message $\mu_{i}$ (is not the target message $\mu^{*}$ and user $ID_{i}^{*}$). Next, *C* keeps a list $L_{S}$ to store these query results. When they obtain the queries from *A*, *C* first checks the list $L_{S}$ whether the result pair $\left(e_{i},c_{i},ID_{i},\mu_{i}\right)$ exists or not. If yes, they send $s_{ID_{i}}$ to *A* as the response of a private key query about $ID_{i}$. If no, they execute the Hash algorithm $H_{2}$ to obtain $\left(c_{i},\mu_{i}\right)$, compute $e_{i}=s_{ID_{i}}c+x$, send $<e_{i},c_{i}>$ to *A*, and record $\left(e_{i},c_{i},ID_{i},\mu_{i}\right)$ in the list $L_{S}$. Here, *A* can execute this query with enough times $q_{S}$ about different identities $\left\{ID_{1},ID_{2},...,ID_{q_{S}}\right\}$ and different messages $\left\{\mu_{1},\mu_{2},...,\mu_{q_{S}}\right\}$.
- *Forge*: *A* utilizes the former received information to generate a valid signature $\left(e_{i}^{*},c_{i}^{*}\right)$ about the target user $ID_{i}^{*}$ and message $\mu^{*}$. Then, they send this forged signature $\left(e_{i}^{*},c_{i}^{*}\right)$ to *C*.
- *Challenge*: *C* utilizes the forking lemma to generate the other valid signature $\left(e_{i}^{**},c_{i}^{*}\right)$ about the target user $ID_{i}^{*}$ and message $\mu^{*}$. Here, *C* utilizes the same selected $x\in D_{\sigma }^{m}$. Then, it has
  (6) $$c_{i}^{*}=H_{2}\left(Ae_{i}^{*}-a_{ID_{i}^{*}}c_{i}^{*},\mu^{*}\right)=H_{2}\left(Ae_{i}^{**}-a_{ID_{i}^{*}}c_{i}^{*},\mu^{*}\right)$$
  With the same Hash function $H_{2}$ and message $\mu^{*}$, it has
  (7) $$Ae_{i}^{*}-a_{ID_{i}^{*}}c_{i}^{*}=Ae_{i}^{**}-a_{ID_{i}^{*}}c_{i}^{*}\;modq$$
  It also has
  (8) $$A\left(e_{i}^{*}-e_{i}^{**}\right)=a_{ID_{i}^{*}}\left(c_{i}^{*}-c_{i}^{*}\right)=0\;modq$$
  Because of $\left|\right|e_{i}^{*}{\left|\right|}_{\propto },\;||e_{i}^{**}{\left|\right|}_{\propto }\leq \;q/4$, it has $\left|\right|e_{i}^{*}-e_{i}^{**}{\left|\right|}_{\propto }\leq \;q/2$ with overwhelming probability. Hence, it can derive a solution $v=e_{i}^{*}-e_{i}^{**}{\;(||v||}_{\propto }\leq \beta )$ for $Z-SIS_{q,n,m,\beta }^{\kappa }$ instance as shown in the following Equation (9).
  (9) $$A(e_{i}^{*}-e_{i}^{**})\;modq=0$$
- *Analyze*: In the common sense, the lattice assumption is not solved with the current highest computing power. This implies that the hypothesis of the former adversary *A* is invalid. Meanwhile, from the former query processes, *A* can create a valid signature $\left(e_{i}^{*},c_{i}^{*}\right)$ with the probability $\frac{\xi }{q_{H_{1}}+q_{H_{2}}+q_{P}+q_{S}}$. With the query times $q_{H_{1}}$, $q_{H_{2}}$, $q_{P}$, and $q_{S}$ increasing, this probability will decrease to 0.

Here, it completes this theme proof, and the proposed PQ-IDS can resist the forgery attack from the malicious adversary.

### 5.3. Non-Repudiation and Traceability

Digital signature plays a role in information protection in most current IoT network applications, and this PQ-IDS scheme utilizes the identity mechanism to achieve secure data exchange among different IoT devices. The proposed security model and PQ-IDS scheme co-guarantee the non-repudiation and traceability of the data-sharing processes in IoT networks.

- *Non-repudiation 1*: The data-sharing transactions are all signed by related workers from the collection to processing and storage. These signatures are signed with the workers’ private keys $s_{ID_{i}}$, and they can be verified by anyone with their public keys $a_{ID_{i}}$. So, the workers cannot deny when the signatures pass the verification.
- *Non-repudiation 2*: The private key is generated by $s_{ID_{i}}\leftarrow Samplepre\left(A,T,a_{ID_{i}},\sigma \right)\in Z_{q}^{m}$, and the user’s public key is generated by $a_{ID_{i}}=H_{1}\left(ID_{i}\right)$ where the $ID_{i}$ is the personal identity, such as identity number, email address, phone number, etc. The personal information guarantees that the signature cannot be denied.
- *Traceability 1*: This identity signature mechanism guarantees the traceability of IoT data exchange processes as the related workers will be traced by opening the corresponding signature when some security incidents occur.
- *Traceability 2*: The blockchain technology provides an immutable public ledger for the records of IoT data storage and operational processes, which guarantees traceability for data sharing in different IoT networks. Once some disputes occur, it can find all the operation records and related operators by opening the signatures.

### 5.4. Post-Quantum Security

As in most current IoT network applications, the cryptographic algorithms inserted in different information systems are generally based on the classical mathematical difficult problems of discrete logarithms and large integer decomposition. These algorithms cannot provide security guarantees for IoT data sharing facing quantum attacks. However, the proposed PQ-IDS scheme in this paper can solve this problem, which is constructed with the lattice assumption as it has strong security against quantum attacks. Meanwhile, to guarantee the traceability of the IoT data-sharing process, this PQ-IDS scheme utilizes the identity mechanism to ensure that every data-processing record is signed with the corresponding operator’s signature. Based on this PQ-IDS scheme, the IoT network applications will have strong security in the future quantum computer age.

## 6. Comparison and Performance

IoT network applications need more efficient algorithms with high computing power and low power consumption. The performance index is the ultimate goal of algorithm design. The efficiency comparison and performance evaluation are given in the following two subsections.

### 6.1. Efficiency Comparison

Key size is the essential item to show the efficiency and practicability of the proposed lattice-based IDS, which also influences the transaction execution in blockchain-based IoT network applications. This section selects five main items of mpk, msk, pk, sk, and sig. for comparison, and Table 3 gives the result. For the mpk of the IoT network, the key sizes with the vector form in Refs. [34,35] are smaller than those with matrix form in Ref. [33] and the proposed IDS. The key sizes of mpk, msk, pk, sk, and sig. in the proposed IDS scheme are equal to or less than those in the other three schemes. These comparison results show that this lattice-based IDS scheme has obvious advantages of efficiency and practicability for data sharing in IoT network applications. Smaller key size leads to more efficient data and transaction verification processes, which is suitable for data sharing in different IoT network applications. Along with the developments of edge computing ability and quantum technology, post-quantum algorithms for IoT network applications will be increasingly in demand.

### 6.2. Performance Evaluation

The performance evaluations are executed on a Windows 11 desktop with Intel Core i7-9700 CPU 3.2 GHz and 16 GB RAM.

(1) Key size evaluation

**Figure 4:**
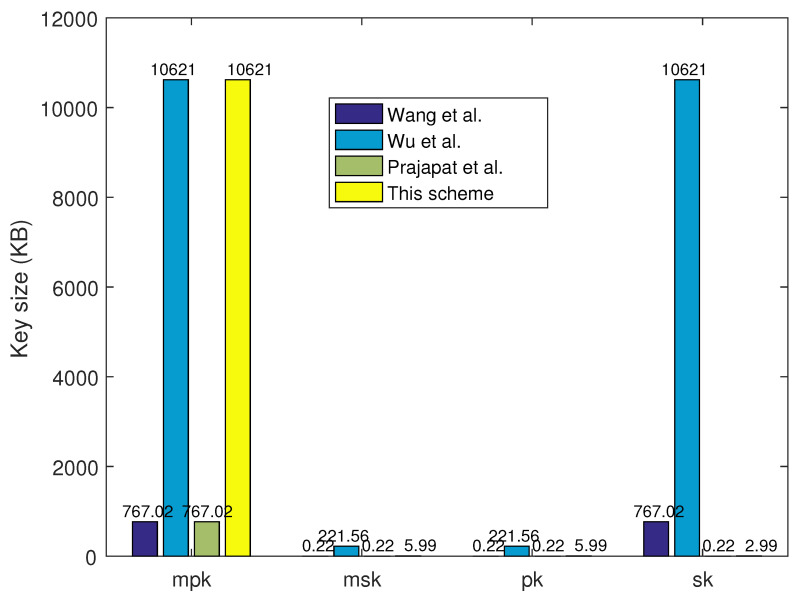
Key size comparison with 80-bit security [33,34,35].

**Figure 5:**
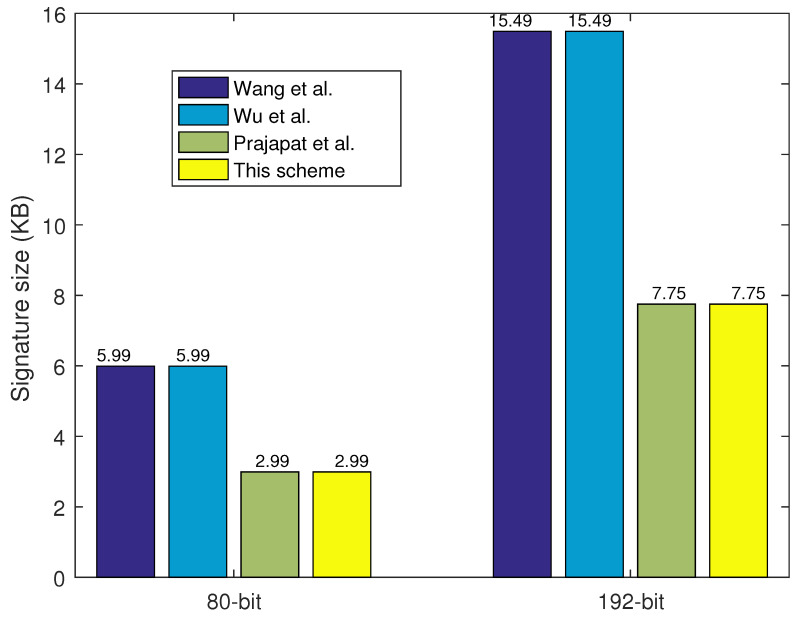
Signature size comparison [33,34,35].

Firstly, the system parameters have been unified. The system parameters are set as n=256, $q=2^{23}$, m=3545, which are equal to the 80-bit security level. The key size comparisons of mpk, msk, pk, and sk with 80-bit security are shown in Figure 4, and the results show that this PQ-IDS scheme is less than or equal to those in the other three schemes. Meanwhile, the signature size comparisons with 80-bit and 192-bit security levels are given as shown in Figure 5. Here, the system parameters are set as n=256, $q=2^{27}$, m=7807, which are equal to the 192-bit security level. The results show that the signature size of the proposed PQ-IDS scheme is no more than that in the other three schemes, and it also shows that the proposed PQ-IDS scheme has good applicability and efficiency for data sharing in different IoT network applications. These results also prove the former theory analyses in Section 5.1. Although the key size of mpk is not small, it can provide a strong security level for IoT network applications facing quantum attacks, and it also can be pre-generated to alleviate the consumption of key generation.

(2) Transaction performance for IoT data sharing

**Figure 6:**
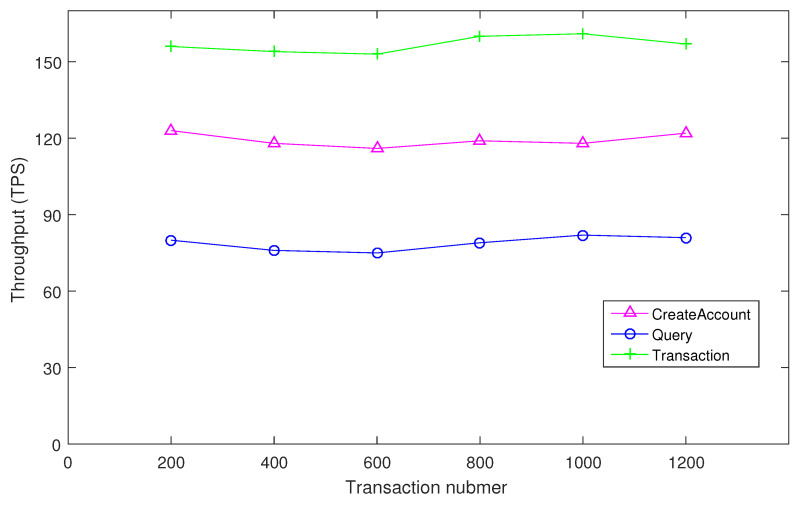
Transaction throughput for IoT data sharing.

**Figure 7:**
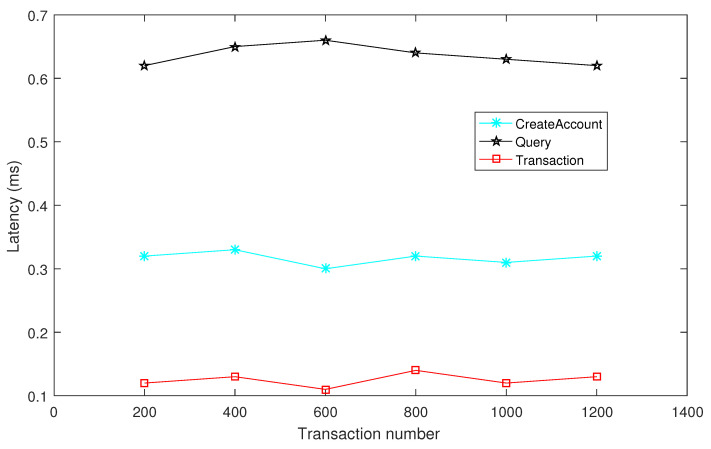
Transaction latency for IoT data sharing.

Under the former stated performance environment, the performance for IoT data-sharing transactions has been given. Here, the throughput and latency are selected for performance, which are the main items for blockchain transactions. Meanwhile, these two items will influence the transaction efficiency. As shown in the following Figure 6 and Figure 7, the performance results of these two items in relation to the “CreateAccount”, “Query”, and “Transaction” are presented. Note that the performance results are the average level of 10 times cross-chain transaction performance, which can eliminate errors from different simulations of the system. From the results, the user registrations have low latency and high throughput, and the establishment transaction amounts remain stable with the number increasing. However, the query times of cross-chain transaction originations have high latency and low throughput compared with the other two items because the IoT data-sharing transactions need high frequency and large quantity.

Above performance evaluations show that the proposed security model is more efficient and practical for data-sharing in IoT network applications, and the proposed PQ-IDS scheme can well support the protection of system data and user privacy.

## 7. Conclusions

Facing security issues in IoT network applications, this paper introduces a PQ-IDS scheme to guarantee data-sharing security among IoT devices in different layers of the IoT network. An IoT network security model is designed first, which divides the IoT network applications into three layers and states the security issues. Meanwhile, a PQ-IDS scheme with lattice assumption has been proposed, which guarantees IoT data-sharing security in the current or future quantum computer age. The identity-based signature mechanism can also provide a traceability mechanism for IoT node failure. Then, the security analysis shows that this PQ-IDS can obtain the security properties of unforgeability, non-repudiation, traceability, and post-quantum security. Moreover, the efficiency comparisons show that the proposed PQ-IDS scheme has smaller key sizes than other schemes, and the transaction performances show that the proposed IoT network security model has good properties. These evaluation results show that the proposed PQ-IDS is efficient and practical for IoT network applications.

The key size of mpk is bigger than that in NTRU-based lattice cryptography, so it is one research purpose to reduce the key size to satisfy the demands of high throughput and low computing power of IoT devices. Meanwhile, as the number of IoT devices increases and the scale of the network increases, the refined control of the access rights and capabilities of different device subjects is an important guarantee for the privacy protection of IoT data and system users. Therefore, access control in a more fine-grained way will be considered more for secure data sharing among different IoT devices or IoT networks in future work.

## Figures and Tables

**Table 1:** Cryptographic algorithm comparisons for IoT network.

Scheme	IoT Network	Main Technology	Limitation	Anti-Quantum
Liu et al. [26]	IoT	ID multi-signature; Threshold Merkle tree; Consortium blockchain	Identity traceability lack	No
Jia et al. [27]	IoT	Certificateless signature; ECC	Identity traceability lack; Centralized management	No
Du et al. [28]	IoT	Certificateless signature; ECC; Security authentication technology	Identity traceability lack; Centralized management	No
Li et al. [29]	IIoT	Aggregate signature; Permissioned blockchain; Smart contract	Identity traceability lack; Data openness; Centralized like management	No
Bao et al. [30]	IIoT	Identity management; Permissioned blockchain; Smart contract	Data openness; Centralized like management	No
Srivastava et al. [32]	IoV	ID multi-signature; Blockchain; Cloud server network	Efficiency to be improved	No
Wang et al. [33]	IoT	Proxy signature; Fixed dimension lattice basis delegation; Preimage sample	Identity traceability lack; Centralized management; Efficiency to be improved	No
Wu et al. [34]	IoT	ID proxy signature; NTRU lattice; Message recovery; Blockchain	Efficiency to be improved	Yes
Prajapat et al. [35]	VANETs	ID aggregate signature; Vehicle pseudo-identities; Cloud storage	Centralized management; Efficiency to be improved	Yes
Sun et al. [36]	XAI network	Path signature; Time series classification	Identity traceability lack; Centralized management	No
This paper	General IoT	ID signature; LWE lattice; Blockchain; Gaussian distribution	Efficiency to be improved	Yes

**Table 2:** System parameter notations.

Notation	Meaning
*n*	Security parameter
*q*	A prime number
*m*	A positive integer with m≥2n⌈logq⌉
*L*	Threshold parameter
σ	The standard deviation
pp	Public parameter
msk	Muster secret key
$ID_{i}$	User i’s identity
$D_{\sigma }^{m}$	The bimodal Gaussian distribution
pk	User’s public key
sk	User’s private key
$H_{1},H_{2}$	The cryptographic Hash function
μ	The message

**Table 3:** Key size comparison.

Schemes	*mpk*	*msk*	*pk*	*sk*	*sig*.
Ref. [33]	mnlogq	$m^{2}logq$	mnlogq	$m^{2}logq$	2mlogq
Ref. [34]	nlogq	$4n^{2}logq$	nlogq	2mlogq	2mlogq
Ref. [35]	nlogq	$4n^{2}logq$	nlogq	2mlogq	mlogq
This PQ-IDS	mnlogq	$m^{2}logq$	nlogq	mlogq	mlogq

## Data Availability

No new data were created or analyzed in this study. Data sharing is not applicable to this article.
